# Does Photobiomodulation Affects CK10 and CK14 in Oral Mucositis Radioinduced Repair?

**DOI:** 10.3390/ijms232415611

**Published:** 2022-12-09

**Authors:** Ariane Venzon Naia Sardo, Maíra Franco Andrade, Anaeliza Figueiredo, Flávia Cristina Perillo Rosin, Luciana Corrêa, Denise Maria Zezell

**Affiliations:** 1 Center for Lasers and Applications, Instituto de Pesquisas Energeticas e Nucelares IPEN-CNEN, São Paulo CEP 05508-000, SP, Brazil; 2 School of Dentistry, University of São Paulo, São Paulo CEP 05508-000, SP, Brazil

**Keywords:** oral mucositis, photobiomodulation, laser, cytokeratin, radiotherapy

## Abstract

The mechanisms of action of photobiomodulation (PBM) in oral mucositis (OM) are not completely elucidated. To enlighten the role of PBM in the evolution of epithelial maturity in OM ulcers, the present study evaluated the effect of PBM with red (λ) wavelength of 660 nanometers (nm) and infrared of 780 nm in radio-induced OM wounds on the tongue of rats, eight and twenty days after irradiation with single dose of 20 Gy. The percentage area corresponding to positive staining for cytokeratin 10 (CK10) and 14 (CK14) proteins was evaluated in the epithelial area of the lesions, using an immunohistochemical technique (IHC), 8 and 20 days after the induction of lesions, and compared with an untreated control group. CK10 was significantly more expressed in the group treated with 660 nm PBM. CK14 did not show quantitative differences between the groups evaluated. However, whereas in the groups treated with PBM, CK14 was already restricted to the basal layer of the epithelium, as expected in healthy epithelia, in control group it was also expressed in upper layers of the epithelium. In this work, PBM was able to improve epithelial maturity of the repaired OM wound, especially in the 660 nm group.

## 1. Introduction

Oral mucositis (OM) is one of the main complications of cancer treatments, which may be initiated by certain chemotherapeutic agents, ionizing radiation for therapeutic purposes in the head and neck region or a combination of these two treatment modalities. [1,2,3]. Regarding radiotherapy conducts, OM begins mainly via indirect damage to DNA of tissue cells, evolving to oxygen free radical generation, through water radiolysis, activation of nuclear factors and consequent tissue degradation process [1,4,5].

OM can manifest in different clinical stages ranging from the presence of a mild erythema to the presence of severe ulcerations. This epithelial discontinuity leaves the patient more vulnerable to opportunistic infections, as well as causing potentially severe painful responses, which may limit the function of the oral cavity. The potential for systemic dissemination of these infections and the general weakness of the patient’s condition caused by OM may increase the hospital costs involved in the hospitalization of this patient [3,6].

Although there is not yet a universal protocol for the clinical management of this condition, the currently available therapies for the clinical management of OM range from pharmacological, using local and systemic anti-inflammatory drugs, oral hygiene protocols, and laser irradiation [7,8]. Low intensity laser irradiation, more recently referred to as photobiomodulation therapy (PBMT), shows success in the clinical treatment of OM, either in cases caused by chemotherapy, radiotherapy, or in cases where OM was caused by the use of combination of these two treatment modalities [9,10,11,12].

The wavelengths widely used for PBM in OM are red and near infrared. However, the literature is heterogeneous with regard to the parameters of the use of low intensity laser, such as wavelength, beam diameter, energy and power densities, and irradiation mode (scan or spot) in the patient are currently consensual in the literature. The mechanisms of action of PBMT in OM are not fully understood and there are few studies reporting cellular effects of PBMT on radiation-induced OM [10,12,13,14,15]. It is more common to find experimental studies in the literature that use chemotherapy and not ionizing radiation to investigate mucositis, due to greater ethical and technical difficulties involving in vivo studies with ionizing radiation [16].

Keratinocytes have filamentous cellular proteins called cytokeratins (CK) in their cytoskeleton. A few decades ago, an identification of these cytoskeletal proteins proved to be important, since their participation in the identification of the pathogenesis of various epithelial disorders was verified [17]. This is because the CK expression occurs in order to change its epithelium according to the change depending on the degree of maturity. This allows for a control or lesser degree of epithelial maturity, according to the pattern of expressed CK. It is suggested that CKs not only participate in the mechanical integrity of the epithelium, but also play a significant role in cell signaling [18]. In the basal layer, CKs are related to epithelial integrity and mechanical resistance, since desmosomal junctions that make up the intracellular connections of the epithelium are linked to the cytokeratin filaments of the cells present [19,20].

CK14 is characteristically observed in epithelial basal layers, where there is greater proliferative activity, as is expected to occur in the tissue healing process, and also in less mature cells. In the suprabasal portion, in more mature cells, the expression of CK14 is less marked. In this uppermost region of the epithelium, CK10 is an example of an abundantly found type of structural protein. CK10 is one of the main CKs that is expressed in the most differentiated layers in keratinized epithelia [17,18,21,22]. Mature and differentiated keratinocytes are capable of, upon aggressive stimuli, secreting factors such as defensins, which participate in the local defense and reflect the competence of the epithelium, in terms of mediating the innate immune response [23,24].

Emphasizing the lack of research models for radioinduced OM and the lack of studies that evidence the action of PBMT in OM at the cellular level, this work aimed to evaluate the PBMT action on OM induced in rats tongue, by single dose of 20 Gray (Gy) from a gamma ray source, in relation to the expression of CK10 and 14 during OM repair. The PBMT groups were treated with red wavelength (λ) of 660 nanometers (nm) and other with infrared of λ = 780 nm, and compared with a control group that did not received any treatment. The epithelial area, market by CK10 and CK14 with immunohistochemistry reaction (IHC), were evaluated in the lesions, and compared with a non-treated group. We used the ImageJ 1.533 p (4 March 2022)^®^ [25] program with Colour Deconvolution 2 Copyright^©^ 2020 Gabriel Landini [26] plugin to calculate the percentage of epithelial area marked by proteins CK10 and CK14.

## 2. Results

The values corresponding to the percentage of epithelial area well-marked by proteins CK10 and CK14, for each of the two groups treated with PBM, with 660 nm laser and 780 nm laser, and for the control group, were submitted to unpaired quantitative analysis, in order to assess whether the area of labeling of each protein varied according to the groups (660 nm, 780 nm or untreated control) and/or with the clinical evolution time of the lesion (8 or 20 days).

### 2.1. Statistical Analysis

The normality test used was the Shapiro–Wilk, which verified the parametric distribution of the data. For this reason and due to the number of groups evaluated, an analysis of variance test (ANOVA) for parametric data was chosen. The test was complemented by Tuckey’s post hoc test. A significance level of less than or equal to 0.05 was considered. All statistical analyses were performed using GraphPad Prism 7.04 (GraphPad Software, San Diego, CA, USA).

### 2.2. Percentages of CK10 Expression by IHQ

**Figure 1:**
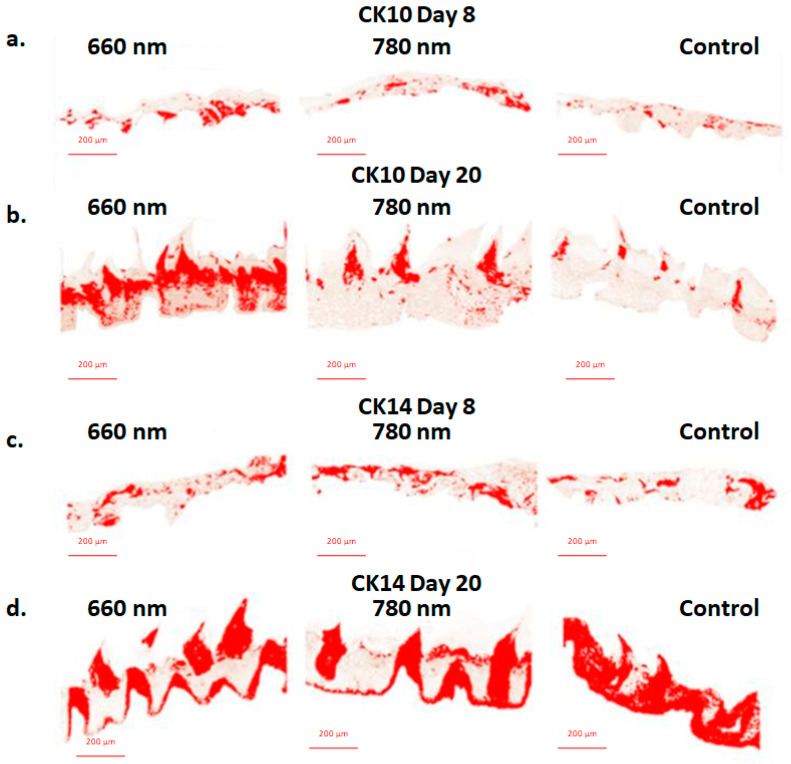
Photograph using the NIS-Elements Advanced Research software AR 4.50.00 (build 1117) Patch 01 64 bit (40×, by optical microscopy (Nikon Eclipse Ti-E, Shinagawa, Japan) and highlighting the area marked by the IHQ, by the Colour Deconvolution 2^®^ plugin on the ImageJ^®^ program. (**a**) CK10 on day 8; (**b**) CK10 on day 20; (**c**) CK14 on day 8; (**d**) CK14 on day 20: note the difference in the distribution pattern of CK14 in the 20-day control group compared to PBMT groups from the same period.

Statistical analysis showed a significant difference in the percentage of epithelial area marked with CK10 for the group treated with 660 nm PBMT on the twentieth day of the experiment (Table 1). This group presented the highest values of epithelial area positively marked by the IHC reaction for CK10 compared to the other experimental groups for all evaluated periods (Figure 1a,b).

### 2.3. Percentages of CK14 Expression by IHQ

There was no statistically significant difference for the values corresponding to the area of the epithelium in which CK14 expression was detected (Table 1). It is noted, however, that the distribution pattern of CK14 on the twentieth day of the experiment is not restricted to the basal layer as seen in the groups treated with PBMT for this period (Figure 1c,d).

## 3. Discussion

The present study illustrates the action of PBMT with λ = 660 nm and λ = 780 nm lasers in relation to the expressions of CK10 and CK14 in lingual epithelium with OM wounds induced by ionizing radiation on the eighth and twentieth day after induction. The PBMT groups had their labeling results compared with each other and with a control group which received no treatment. According to our literature review, this is the first study to illustrate the effects of PBMT with red and infrared lasers on ionizing radiation-induced MO by evaluating the expression of CK10 and CK14.

The induction of the clinical picture of ulceration caused by OM, in the present study, was carried out by means of irradiation with 20 Gy through a gamma ray source. Such dosimetric parameter is within what the literature points out as the range of energy values necessary for the development of OM ulcerations in an animal model [27,28,29]. Due to technical difficulties related to the induction of OM by ionizing radiation for research purposes, the most frequent OM models in the literature involve the use of chemotherapeutics, and not radioactive sources [16]. This fact reinforces the importance of outlining studies that mimic the action of radiotherapy in OM.

The clinical periods of euthanasia of the animals were chosen based on the clinical time points. On the eighth day of the experiment, the ulcerated lesions reached their peak of clinical evolution, a fact that was observed in the experimental phase of the pilot project that preceded this study. The twentieth day of the experiment and the last period of sample collection in this study was chosen based on the fact that the OM lesions on day 20 of the experiment were completely closed in all animals in the sample, as expected by the lesion’s natural evolution course [30]. In addition, in this OM model, the onset and end of reepithelization process are at 8 and 20 days, respectively, time points that are adequate for the analysis of CKs expression.

By using the same physical parameters of irradiation, our study allows us to clearly compare the action of red and infrared lasers. The parameters for using PBMT as a therapeutic modality in OM remain heterogeneous and it is still not possible to accurately outline a dosimetry protocol and a consensus on the wavelengths to be used. In 2019, a systematic review highlighted the high variability in energy density parameters for therapeutic protocols in OM, whose energy density ranged from 1 to 70 J/cm^2^ per irradiation point and the device power from 15 to 125 mW. The authors mention a large number of studies involving PBMT in OM being discarded from their systematic review, for not correctly reporting the laser dose parameters used [15]. In this work, the laser irradiation parameters of 660 nm and 780 nm emitted the same power of 30 mW and energy density of 7.5 J/cm^2^ per point (see Table 2).

Similarly to De Castro [31], this work used the percentage of tissue area positively marked with the IHQ, to compare the expression of CK10 and CK14 proteins, between groups of different clinical times of OM lesions, by means of colorimetric deconvolution. The plugin used was Color Deconvolution 2, on ImageJ software (ImageJ, U. S. National Institute of Health, Bethesda, MD, USA), as a means of separating the area corresponding to the marking by IHQ in the images. Thus, it was possible to obtain the virtually assisted separation of the three color spectra that make up the original coloration of the IHC reaction: brown color of diaminobenzidine (DAB), hematoxylin blue color, and residual colors [32]. So, images containing the brown color of DAB were used, which is the chromogen of the IHC reaction, corresponding to the labeling of the proteins here investigated.

Therefore, the analysis carried out in this work was limited to quantifying the area of the epithelium that was or was not marked by staining, after verifying the quantification made by the post deconvolution software. This dichotomy in the analysis, limited to pointing out what is or is not marked, is in accordance with the fact that the optical density of the DAB marking in the IHC does not follow the Beer–Lambert law in the deconvolution image. In other words, it is not true to relate the protein concentration to the intensity of the brown tone obtained in the spectral deconvolution image [26].

In the present study, significant differences were found on the twentieth day of the experiment in relation to the expression of CK10 for the group treated with 660 nm laser, which presented a significantly higher percentage of positive staining per epithelial area, compared to the other groups in the experiment, such as it can be seen in Figure 1. In fact, CK10 is associated, in samples of normal lingual epithelium, with extracts of supra basal cells, with a greater degree of maturity and differentiation [17,18,22]. Reinforcing our results, the work of Antunes and his collaborators [33] verified that PBM with red laser is able to stimulate the expression of genes related to the differentiation of human keratinocytes from an in vivo study that evaluated the PBMT response in OM. In addition, it is known that the red laser has a more superficial action than in depth when in contact with tissue [34], which justifies its greater effectiveness in relation to the other groups in terms of the positive epithelial response found here.

In accordance with the results of this study, De Castro [31] reports that the increase in CK10 expression is directly associated with better clinical and histomorphological quality of epithelial repair in rat skin, in which surgical wounds were treated with PBMT with 660 nm laser. Moreover, Sperandio [32] found a higher expression of CK10 on epithelial from skin wounds in rats treated by 660 nm PBMT, and associates this with increased epithelial maturity and migration velocity of keratinocytes. The potential increase in epithelial maturity and differentiation stimulated by the red laser could mean a positive gain in the functional competence of keratinocytes [23,24].

No statistical differences on CK14 staining area was found between the groups in this study. Nonetheless, in healthy epithelial samples, CK14 is exclusively expressed in basal layers cells and in this study, this protein appears, on twentieth day control group, expressed by superficial layers, even with no more tongue ulceration. This altered expression pattern is associated in the literature with epithelia whose repair has not yet taken place [32,35].The few studies published to date that evaluated the expression of this protein in OM lesions found that CK14 and CK5 found positively expressed also in upper epithelial extracts not limited to the basal layer, as in healthy epithelia [21,29]. In contrast, the PBMT groups on day 20 shows expressions of CK14 restricted to the basal layers, as expected from normal epithelia.

The findings of this study suggest that PBMT with red and infrared wavelengths in the parameters used here was able to normalize the distribution of CK14 in the epithelium previously submitted to OM lesions by ionizing radiation, as it restricted the presence of less differentiated cells with high proliferative power to the basal layer, which is, according to literature, observed in samples of healthy epithelium. Furthermore, the data in this study allow us to suggest that the red laser was significantly associated with increased CK10 expression in the samples evaluated here, on the twentieth day of the experiment. Thus, PBMT is able to positively collaborate with the degree of epithelial maturity in the pair process of OM lesions induced by ionizing radiation.

## 4. Materials and Methods

### 4.1. OM Induced by Ionizing Radiation

We use 34 male Wistar rats with approximate body mass of 350 g, from the IPEN animal facility (Institutional Ethics Committee Approval 94/11/CEUA-IPEN/SP). The animals were kept in cages containing up to 03 animals each, receiving water and pelleted Nubivac^®^ commercial feed at will, with 12 h light/dark cycles. 

Prior to the start of the rat experiment, the Cobalt—^60^Co panoramic source dosimetry was performed to obtain the dose rate and amount of lead shielding required. An online FSH-260 silicon photodiode dosimeter was positioned at the source and associated with an electronic outside the radiation chamber. The air dose rate was used for dosimeter calibration, and then the distance from the source and the amount of lead required for radiation shielding were measured.

This phase consisted of the ionizing radiation dosimetry required to induce OM and it was based on previously published study [36]. From the dosimetry with the photodiode, it was found that the thickness of 10 cm of lead was the best shielding for the body of animals 20 cm away from the source. At this distance, a dose rate of 0.60 Gy/min was obtained, determining an initial exposure time of 33 min, totaling 20 Gy delivered to the tissue. For this, three blocks of lead of 5 cm thick were used to protect the trunk and lower limbs of the animal, aiming to expose only the head region for the irradiation procedure.

To perform the containment of animals during this experiment was used chemical anesthesia, intraperitoneally, with the combination of drugs ketamine (DCB 01937), dose of 75 mg/kg and xylazine (DCB 09207), dose of 10 mg/kg [37]. They were then radially arranged to the ionizing radiation source, with a focal length of 20 cm, with a panoramic ^60^Co source irradiator.

### 4.2. PBMT Experimental Design

After ionizing irradiation, the animals were randomly divided into three groups, two being treated by PBMT, in a laser group of λ = 660 nm and in the other laser group of λ = 780 nm. The irradiation parameters are listed in Table 2. The third group consisted of animals that were not submitted to any therapeutic modality, this being the study control group. However, this group was subjected to the same manipulation and procedures as those of PBMT, in order to avoid factors that could differentiate the clinical results between the groups: chemical containment, irradiation support, type of feed, and housing were mimicked contact with the disconnected laser delivery part for the same period of 10 s per point. The animals were further divided into three periods for clinical evaluation, 8 and 20 days. The number of rats in each group is described in Table 2.

Animals were anesthetized according to the protocol previously described in this text, with the combination of drugs ketamine (DCB 01937), dose of 75 mg/kg and xylazine (DCB 09207), dose of 10 mg/kg, placed in an appropriate support, specially developed for this project, and then submitted to GaAlAs diode laser irradiation (MMOptics^®^, São Carlos, SP, Brazil) of the tongue every two days. Three points of irradiation on the back and two points of irradiation on the lingual belly were chosen to cover the entire exposed surface of the organ without inducing iatrogenic trauma.

### 4.3. IHQ Analysis

About3μM thick sections disposed in silanized glass slides were deparaffinized at 60 °C. The sections were rehydrated in xylene, placed in decreasing strengths of alcohol and, finally, in water. Material was immersed in 1% bovine serum albumin for one hour for blocking the non-specific sites. Then, sections were incubated for 1 h at room temperature, with primary anti-CK10 antibody (at a concentration of 1:100) and anti-K14 (1:400). To evidence the region positively marked with proteins, the chromogen diaminobenzidine (DAB) was used (Dako Liquid DAB Plus, Dako Corporation, Carpinteria, CA, USA) and counter stained with Mayer’s hematoxylin. After completing this reaction step for evidencing CK10 and CK14 proteins by DAB staining, the slides were photographed using the NIS-Elements Advanced Research software, by optical microscopy (Nikon Eclipse Ti-E, Shinagawa, Japan). 

The area corresponding to the epithelium of each of these sections was then selected with selection tool available in the ImageJ software (ImageJ, U. S. National Institutes of Health, Bethesda, MD, USA). The same program obtained, after this delimitation, the total value of the epithelium area. Thus, an image was obtained that contained only the area of the epithelium that appeared in the field, and then evaluated. After this process of selection of the epithelial area, and obtaining its numerical values, the analysis of DAB staining was carried out using the Color Deconvolution 2 plugin, whichspecificallyseparatestheimagecorrespondingtotheareamarkedwiththe DAB dye and allows, with that is, the quantification of the tissue area in which the protein in question is expressed [26]. The total area of the epithelium was related to the area marked by each of the proteins and a percentage value of the area of epithelium positively marked with the proteins studied here was obtained: [31] % epithelium positively marked = (Area marked by IHQ reaction ÷ Total Area) × 100.

## 5. Conclusions

The data in this study allow us to suggest that the red laser was significantly associated with increased CK10 expression in the samples evaluated here, on the twentieth day of the experiment. Furthermore, PBMT with red and infrared wavelengths in the parameters used here was able to normalize the distribution of CK14 in the epithelium previously submitted to OM lesions by ionizing radiation, as it restricted the presence of mitotic cells with high proliferative power to the basal layer, which is, according to the literature [17,18,22], observed in samples of healthy epithelium. Thus, PBMT is able to positively collaborate with the degree of epithelial maturity in the repair process of OM lesions induced by ionizing radiation.

## Figures and Tables

**Table 1:** Table containing the mean and standard error of the percentage corresponding to the epithelial area marked by CK10 and CK14, according to the experimental groups.

Group	Mean ± SEM CK10	Mean ± SEM CK14
660 nm day 8	8.69 ± 1.271	23.82 ± 2.22
660 nm day 20	38.01 ± 1.228 ^1^	32.11 ± 6.485
780 nm day 8	7.32 ± 1.099	19.8 ± 1.551
780 nm day 20	10.35 ± 1.822	28.88 ± 4.237
Control day 8	4.315 ± 1.269	26.82 ± 6.064
Control day 20	5.87 ± 0.836	39.12 ± 5.972

^1^ Different results for a significance level ≤ 0.05, according to ANOVA test with post hoc Tukey.

**Table 2:** Distribution of animals in the experimental groups, according to the days of clinical evaluation and description of irradiation parameters.

Groups	Day 8	Day 20	Total Rats in Each Group
λ = 780 nm, 30 mW, 7,5 J/cm^2^, 10 s, Spot size = 0.04 mm; Irradiation = 48/48 h	07	07	14
λ = 660 nm, 30 mW, 7,5 J/cm^2^, 10 s, Spot size = 0.04 mm; Irradiation = 48/48 h	07	05	12
Control	04	04	8

## Data Availability

The data that support the findings of this study are available from the corresponding author, [D.M.Z.], upon reasonable request.
